# Enzootic *Trypanosoma cruzi* infection by *Rhodnius prolixus* shows transmission to humans and dogs in Vichada, Colombia

**DOI:** 10.3389/fcimb.2022.999082

**Published:** 2022-10-18

**Authors:** Omar Cantillo-Barraza, Cesil Solis, Alexander Zamora, Rafael Herazo, María Isabel Osorio, Edilson Garcés, Samanta Xavier, Ana María Mejía-Jaramillo, Omar Triana-Chávez

**Affiliations:** ^1^ Grupo Biología y Control de Enfermedades Infecciosas - BCEI, Universidad de Antioquia (UdeA), Medellín, Colombia; ^2^ Programa de Promoción, Prevención y Control de Enfermedades Transmitidas por Vectores, Secretaría de Salud del Vichada, Puerto Carreño, Colombia; ^3^ Laboratory of Trypanosomatid Biology, Oswaldo Cruz Institute, Fundaçao Oswaldo Cruz (FIOCRUZ), Rio de Janeiro, Brazil

**Keywords:** Chagas disease, Colombia, *Rhodnius prolixus*, *Triatoma maculata*, *Trypanosoma cruzi*, *Didelphis marsupialis*

## Abstract

**Background:**

*Rhodnius prolixus* is considered the most relevant *Trypanosoma cruzi* vector in Colombia and Venezuela due it is responsible for domestic transmission in both countries. However, a wild population of this species is distributed in the eastern plains of the Orinoco region and Amazonia jungle, where its epidemiological importance has not been sufficiently elucidated. This study aimed to assess epidemiological parameters of *T. cruzi* transmission in the Department of Vichada, Colombia.

**Methods:**

We determined the characteristics of *T. cruzi* transmission using entomological studies in domestic and sylvatic ecotopes. We analyzed the *T. cruzi* infection in triatomine insects, identified blood meal sources, and conducted a serological determination of *T. cruzi* infection in scholar-aged children, domestic dogs, and wild hosts.

**Results:**

Fifty-four triatomine bugs, 40 *T. maculata* and 14 *R. prolixus* were collected in peridomestic and sylvatic ecotopes. Infected *R. prolixus* was observed in La Primavera, Santa Rosalia, and Cumaribo municipalities. All the *T. maculata* bugs were not infected. Serological analysis indicated that two of 3,425 children were *T. cruzi* positive. The seroprevalence in domestic dogs was 10,5% (49/465). Moreover, 22 synanthropic mammals were sampled, being *Didelphis marsupialis* the most common. TcI genotype was detected in seropositive dogs, *R. prolixus*, and *D. marsupialis.*

**Conclusion:**

The present work describes extra domestic *R. prolixus* and *D. marsupialis* in a sylvatic *T. cruzi* transmission cycle with transmission to humans and domestic dogs in Colombia’s Vichada Department.

## 1 Introduction

Chagas disease is a zoonosis caused by the protozoan hemoflagellate *Trypanosoma cruzi*, which is transmitted to humans mainly by insects of the triatominae subfamily (Hemiptera: Reduviidae) (Chagas, 1909). About 8 million people are currently infected with the parasite, and at least 10,000 deaths related to this disease occur each year (WHO, 2015). *T. cruzi* presents enormous genetic diversity and has been divided into six Discrete Typing Units (DTUs) called TcI to TcVI (Zingales et al., 2012). More recently, another genotype found in bats called TcBat was also reported (Ramirez et al., 2014). These genotypes are frequently associated with different clinical manifestations, geographical distribution, and transmission cycles (domestic, peridomestic, and sylvatic). TcI is the most widely distributed DTU in Colombia (Cura et al., 2010; Guhl and Ramirez, 2013).

In Colombia, 27 species of triatomines have been reported in 423 municipalities within 31 departments, where infected species participate in the domestic, peridomestic, and enzootic transmission cycles (Guhl, 2007a; Guhl et al., 2007; Ramírez et al., 2012; Cantillo-Barraza et al., 2014; Rendon et al., 2015). *Rhodnius prolixus* is the *T. cruzi* primary vector species in this country due to its domiciliation (Guhl et al., 2007; Guhl, 2007b). A national program for the interruption of the transmission by intradomestic populations of this species has been designed and implemented (Ministerio de la protección social, 2012). However, in the eastern plains of the Orinoco and Amazon regions, *R. prolixus* sylvatic populations in *Attalea butyracea* and oil palm plantations (*Elaeis guineensis*) have been described as part of enzootic transmission (Guhl et al., 2009; Rendon et al., 2015).

Approximately 2% of the Colombian population is infected with this parasite Llau et al., 2019; Olivera et al., 2019). However, there are still large geographical areas where the proportion of infected people has not been explored (Llau et al., 2019). Domestic and wild mammals, which play a fundamental role in local transmission in unstudied areas, are also poorly defined (Guhl et al., 2007; Rodríguez-Monguí et al., 2019). In this country, *Canis lupus familiaris* and *Didelphis marsupialis* are mammalian species with the highest epidemiological importance in Chagas disease (Guhl and Ramirez, 2013; Rodríguez-Monguí et al., 2019). Domestic dogs actively participate in the domestic cycle and play essential roles as synanthropic mammals (Ramírez et al., 2012; Rodríguez-Monguí et al., 2019; Cantillo-Barraza et al., 2020a). However, *D. marsupialis* is the species with the highest prevalence, and *T. cruzi* infection with an active role in the sylvatic transmission cycle was previously reported (Rodríguez-Monguí et al., 2019; Cantillo-Barraza et al., 2020a).

The Vichada department, located in the Orinoco region on the border with Venezuela, has ecological and geographical features appropriated to *T. cruzi* transmission. The north and west zones contain oil palm plantations, and the south and southeast are comprised of the amazon jungle (Tropical humid biome of the Amazon). The Vichada department is surrounded in its northern section by Chagas endemic departments of the eastern Colombian plains such as Casanare, Arauca, and Meta, and the Venezuelan state of Apure to the west. All these regions have been reported with domiciliation of *R. prolixus* (Feliciangeli et al., 2007; Guhl et al., 2007; Ceccarelli et al., 2018). Also, the jungle subregion of Vichada is a neighbor of the Guainia department in Colombia and the Amazonas state in Venezuela, where non-domiciled vectors such as *Panstrongylus geniculatus, P. lignarius*, and extra domestic *R. prolixus* have been observed (Solis-Medina et al., 2021). Although in the Vichada department, *R. prolixus* and other non-domiciled vectors, such as *T. maculata*, *P. geniculatus, Erathyrus mucrunatus*, and *P. lignarius*, have been reported, few studies related to its relevance in the parasite transmission have been carried out. Therefore, the present study aimed to a) estimate the *T. cruzi* seroprevalence in children, dogs, and synanthropic mammals; b) identify vector triatomine and calculate the natural infection rate, c) describe the blood-meal source in triatomines bugs; and d) determine the *T. cruzi* genotypes present in the region.

## 2 Material and methods

### 2.1 Study area

The Vichada is the second largest Department in Colombia, with an area of 102,242 km^2^ and four municipalities with altitudes between 53 to 180 m.a.s.l (meters above sea level). This study was conducted between 2016 to 2017 on Colombian’s eastern plains of the Orinoco Region and Amazon jungle (2°43’37” and 06°21’18’’N) (67°24’24” and 71°05’28’’W). Three-quarters of the Department are savannahs, and the rest is a tropical jungle (Gallego Gallego, 2018). The weather is tropical, with defined dry (from January to March) and rainy (from April to December) seasons. An average annual temperature of 27°C and rainfall of 2,688 mm (Edgar et al., 2007). This Department is populated by around 73,702 inhabitants (30,660 in urban and 43,042 in rural areas, respectively). Indigenous people constitute the majority of the population, with five indigenous ethnicities present in this Department: Sikuany, Curripacos, Piaroas, Puinaves, and Piapocos (Ministerio del interior (2022).

**Figure 1:**
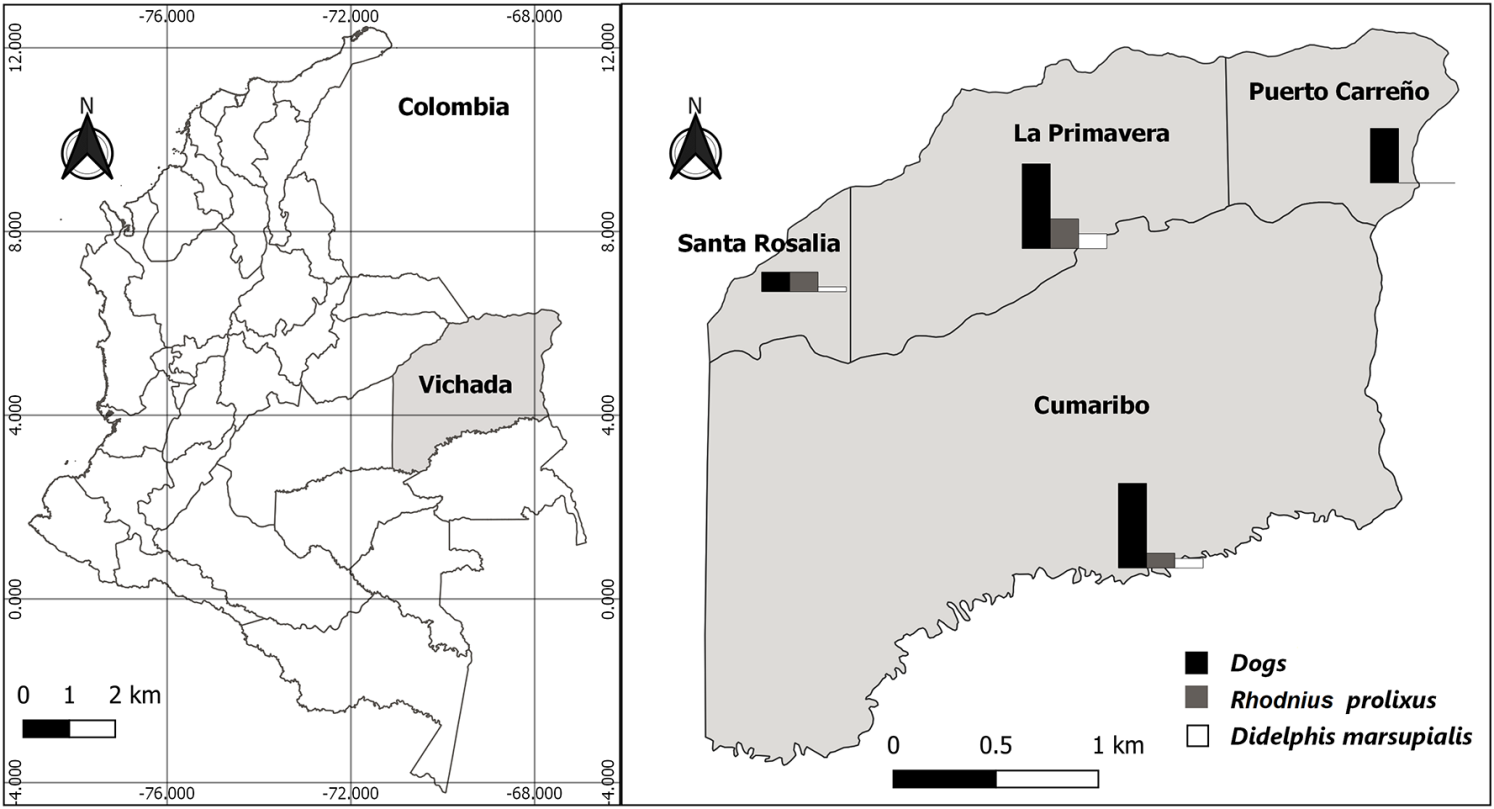
Study area and seroprevalence to *Trypanosoma cruzi* in dogs, frequency of infection of triatomine bugs, and *Didelphis marsupialis* distributed by each municipality in Vichada Department, Colombia.

The survey was carried out in four municipalities in the Department: Puerto Carreño (PC), La Primavera (LP), Santa Rosalia (SR), and Cumaribo (C). The first three are savannas and the last with savannas and jungle (**Figure 1**).

### 2.2 Entomological samples

#### 2.2.1 Intradomestic and peridomestic survey

Five entomological surveys, each around twenty days, were performed during the study period in the four municipalities. All procedures were carried out by technicians of the University of Antioquia, following the National Protocols of Entomological Surveillance. In brief, outdoor and indoor triatomine niches were searched for 30 minutes; flashlights were used to help see cracks and crevices throughout the fabric of buildings, behind pictures on the walls, furniture, in closets, and, especially, under bedding material. All households in neighborhoods with previous reports of triatomine bugs were visited in the urban area. In rural areas, all dwellings were inspected (**Table 1**).

**Table 1:** Summary of capture and *T. cruzi* infection in triatomine bugs, seroprevalence in dogs, synanthropic mammals, and human *T. cruzi* infection in the study area: Puerto Carreño, La Primavera, Santa Rosalia, and Cumaribo.

			Domestic Dogs		Synanthropic mammals				Extradomestic Triatomine insects		Human screening	
					*D. marsupialis*		Other species		*R. prolixus* collected (% infected)	*T. maculata* collected (% infected)		
Municipality	Locality	Evaluated Houses	n	Infected (%)	n	Infected (%)	n	Infected (%)			n	% infection
Puerto Carreño	Urban Area	139	34	1 (2.94)	2	0	2^A^	0		40 (0%)	616	0
	Casuarito	40	16	1 (6.25)							133	0
	Garcitas	20	16	9 (56.25)	2	0					10	0
La Primavera	Urban Area	125	62	14 (22.6)	6	2 (33.3%)	1^B^	0	5 (100%)		650	0
	Santa Cecilia	10	3	2 (66.6)	1	1 (100%)			2 (50%)		39	0
	San Theodoro	15	8	1 (12.5)							94	0
Santa Rosalía	Urban Area	95	31	2 (6.45)	2	1 (50%)	1^C^	0	4 (100%)		210	0
	Pavanay	10	6	2 (33.3)							25	0
Cumaribo	Urban Area	84	62	6 (9.6)	1	1 (100%)			3 (100%)		609	0
	Amanaven	13	20	1 (5)							9	0
	Guayabal	15	10	1 (10)							14	0
	Pueblo Escondido	18	17	0							58	0
	Pueblo Nuevo de Zama	29	15	0							62	0
	San Luis de Zama	12	7	0							34	0
	Sarrapia	24	29	1 (3.44)							151	0
	Puerto Nariño	10	13	1 (7.69)							64	0
	Barranquilla	10	7	0							21	0
	Cejal	12	7	0							14	0
	Chocón	14	6	0							134	1
	Cumaral	18	6	0							136	0
	Morichal	15	10	1 (10)							27	0
	Guerima	35	27	2 (7.4)	1	0					61	0
	Morocoto	10	8	1 (12.5)	2	1 (50%)					53	0
	Chaparral	12	6	0							79	0
	Palmarito	32	22	1 (4.54)							143	1
	Tuparro	30	17	2 (11.7)			1^A^	0			111	0
Total		847	465	49 (10.33)	17	5 (29.4%)	5		14	40	3,425	2

^A^Rattus rattus. ^B^Potus flavus. ^C^Proechymis semispinosus.

#### 2.2.2 Extradomestic survey

Palm trees (*n*=60), *A. butyracea* (Zuleta-Duenas et al., 2017)*, E. guineensis* (Feliciangeli et al., 2007), and *Maurita flexuosa* (Guhl et al., 2007) located inside the studied town or found less than 500 meters from humans’ livings in peripheral areas were tested for triatomines with five live bait traps per palm/night. Additionally, dry, green leaves, organic debris, interfoliaceous meshes, and bracts were examined for the presence of triatomines with the help of a ladder. Insects were transported to the laboratory, registered, and identified using taxonomic keys (Lent and Wigodzinsky, 1979).

### 2.3 Blood-meal source determination

Genomic DNA was extracted from 200 µL of triatomine feces using the Genomic DNA purification kit (DNeasy Blood & Tissue kit Qiagen, Germantown, USA) following the manufacturer’s instructions. The DNA was subjected to a conventional PCR targeting the cytochrome b (cytb) gene vertebrates to identify blood-meal sources. The PCR was performed in a final volume of 25 µL containing 40-50 ng of genomic DNA, buffer 1X, 0,2mM of dNTP, 3mM MgCl_2_, 0,4 µM of each primer (cytbF and cytbR), and 0,025 U Taq polymerase (Invitrogen, California, USA). The amplifications were performed using a thermal cycler of initial denaturation at 95°C for 4 min, followed by 36 cycles at 95°C for 30 s, 60°C for 50 s, and 72°C for 40 s; and a final extension at 72°C for 5 min (Peña et al., 2012). Positive PCR products were purified and sequenced in both strands using the Sanger method at Macrogen, Seoul, South Korea. The sequences obtained were compared with sequences deposited in GenBank using the BLASTN search to identify the host species associated with triatomines.

### 2.4 Mammal host samples

#### 2.4.1 Dog samples

Sampling was carried out in 26 places located in the four municipalities. The sample size was calculated using Epi info 7.0 (www.cdc.gov), considering a population of ~7,000 dogs in the Vichada department, a 22,5% probability of being infected according to (Jaimes-Dueñez et al., 2017), a confidence interval of 95%, and a margin of error of 4%. The estimated sample size was 435, but it was increased by 10% to compensate for sampling error. A non-probabilistic sample was conducted for taking blood samples of canines through a house-to-house strategy. Inclusion criteria for selected dogs were as follows: (i) born and grown in the study area, (ii) dogs with a recognizable owner, (iii) available information about the animal’s history (e.i. age, site of repose, often feeding and health).

Blood samples were taken from dorsal-tibial or radial veins under minimal stress with the owner’s help. The samples were centrifuged, serum was stored at -20°C for serological assays, and the remaining was used for DNA extraction and molecular diagnosis (see below).

#### 2.4.2 Dogs serological diagnostic

Detection of anti - *T. cruzi* antibodies was conducted using the Indirect Immunofluorescence Antibody Test (IFAT) and the Enzyme-Linked Immunosorbent Assay (ELISA, Bio-Manguinhos, FIOCRUZ, Rio de Janeiro, RJ, Brazil). The cut-off criteria for a reactive test were a titer of 1/40 for IFAT and optical absorbance ≥ 0,200 (mean +/- 3 SD) for the ELISA test. Animals were defined as seropositive when samples were reactive for both IFAT and ELISA tests. To evaluate cross-reactions and mixed infection by *T. cruzi* and *Leishmania* spp., dog sera were also assayed for antigens derived from a mixture of *L. infantum* and *L. panamensis* using IFAT and the Rapid Test for Diagnosis of Canine Visceral Leishmaniasis (CVL) (TR DPP^®^, Bio-Manguinhos, FIOCRUZ, Rio de Janeiro, RJ, Brazil) (DPP).

The IFAT cut-off value adopted for *T. cruzi* infection was 1/40 when the IFAT result for *L. infantum* was lower than 1/40, and the DPP result was negative. On the other hand, for *L. infantum* positive dogs, positive *T. cruzi* infection was considered only when the IFAT titer was 1/80 or higher. For *L. infantum* infection, the adopted IFAT cut-off value was 1/40 when the infection was confirmed by DPP and 1/80 when the DPP assay was negative.

#### 2.4.3 Synanthropic mammal capture and diagnostic

Synanthropic mammals were captured using traps (Tomahawk) baited with a mixture of peanut, banana oat, and fish. At each locality, the traps were set for three nights in the forests where palms were sampled and were distributed in linear transects, with capture points established every 20 mts. To detect *T. cruzi*, trapped animals were anesthetized (ketamine, 100mg/kg), and their blood was collected by cardiac puncture. Two tubes containing NNN medium, covered with a LIT overlay, were inoculated with 0,2 mL of blood from each specimen. They were examined for epimastigote forms presence weekly for three months. The remaining samples were stored for DNA extraction and molecular diagnosis (See below).

### 2.5 *Trypanosoma cruzi* infection in humans

With the previous written informed consent of one or both parents and following the University of Antioquia Ethics committee (08–012–185), blood sampling was obtained on 3,425 students between 5 to 20 years old. All schools in the study area were chosen to sign the written informed consent and blood sampling. Approximately 5 ml of whole blood was collected by venipuncture centrifuged, and the obtained serum was stored under refrigeration until further processing.

#### 2.5.1 Serologic analysis

All participants were evaluated by two Enzyme-Linked Immunosorbent Assay (ELISA) tests with different principles, according to the recommendations of the National Institute of Health, Colombia. Anti - *T. cruzi* IgG was detected by two serological tests: (i) for all samples, one initial screening by ELISA test (enzyme-linked immunosorbent assay*)* based on crude parasite extract using two *T. cruzi* isolates (I.RHO/CO/00/CAS-15.CAS; I.TRI/CO/03/MG-8.MAG) were used. The optical density (OD) values of previously confirmed positive and negative controls were used to define the limits for seropositivity and seronegativity in this assay. OD values higher than 2 SD of the OD average for negatives control were considered ELISA-positives. (ii) ELISA test with recombinant antigens (Dia Pro Diagnostic Bioprobes *T. cruzi*-AB), following the manufacturer’s instructions. The incongruent samples were analyzed by one additional serological test: Indirect Immunofluorescence Assay (IFAT). The incongruent samples, reactive to at least one of two complementary tests, were considered positive.

### 2.6 *Trypanosoma cruzi* detection in triatomines, domestics dogs, and synanthropic mammals

Using parasitological and molecular methods, all triatomines collected were evaluated for *T. cruzi* infection. Feces were obtained by abdominal compression, diluted in 300 μL of sterile PBS pH 7.2, and used for DNA extraction. A 10 µL aliquot was examined under an optical microscope at 400X for flagellated forms. The remaining samples were used for genomic DNA extraction with a DNA purification kit (DNeasy Blood & Tissue kit Qiagen, Germantown, USA) and *T. cruzi* genetic typing (see below).

Total DNA extracted from feces of captured triatomines and blood from domestic dogs and synanthropic mammals was used for *T. cruzi* molecular diagnosis. All DNA preparations were screened to test for *T. cruzi* using a conventional PCR targeting satellite DNA (Moser et al., 1989). The PCR was performed in a final volume of 25µL containing 40-50 ng of genomic DNA, 1X of a buffer, 0,04 mM of dNTP, 1,5 mM MgCl_2_, 0,4 µM of each primer (TCZ1 and TCZ2), and 0,05 U of Taq polymerase (Invitrogen, California, USA). The thermal cycling conditions were as follows: pre-heating at 95°C for 15 min, 40 cycles at 95°C for 10 s, 55°C for 15 s, and 72°C for 10 s in a thermal cycler. Positive *T. cruzi* samples were analyzed for molecular discrimination of *T. cruzi* DTUs based on the amplification spliced leader intergenic region (SL-IR) gene using the primers TCC, TC1, and TC2, previously reported (Souto et al., 1996). The PCR was performed in a final volume of 25µL containing 40-50 ng of genomic DNA, 1X of a buffer, 0.25 mM of dNTP, 2 mM MgCl_2_, 0.4µM of each primer, 0.05 U of Taq polymerase (Invitrogen, California, USA). The thermal cycling conditions were as follows: pre-heating at 94°C for 5 min, 35 cycles at 94°C for 30 s, 55°C for 30 s, and 72°C for 45 s in a thermal cycler, and a final extension at 72°C for 5 min. Amplification products were run on a 1.5% agarose gel stained by ethidium bromide and visualized under UV light. PCR products were purified and sequenced using the Sanger methodology at Macrogen sequencing service, Seoul, South Korea, for direct sequencing of the SL-IR region.

## 3 Results

### 3.1 The natural infection rate in triatomines

Fifty-four triatomine bugs, 40 *T. maculata* and 14 *R. prolixus* were collected. *T. maculata* was captured in Puerto Carreño in chicken coops associated with the peridomestic area. However, no *T. cruzi* infection was registered in this species. *R. prolixus* was collected in La Primavera, Santa Rosalia, and Cumaribo in *A. butyracea* and *Maurita flexuosa palms* (**Table 1**). *T. cruzi* infection in this species was found in all municipalities. On the other hand, 847 households were visited, none of which triatomine bugs were found inside the home (**Table 1**).

### 3.2 Blood-meal sources identification

Eight *R. prolixus* and five *T. maculata* were analyzed for blood meal identification by PCR and sequencing of the cyt B gene. One of the three amplified sequences of *R. prolixus* showed an identity of 99.9%, with sequences derived from chicken (*Gallus gallus*). The remaining two showed the highest identity, with sequences derived from the *Ceracris kiangsu* reptile (**Table 2**). No amplification was found in *T. maculata.*

**Table 2:** Stage, capture site, blood meals, GenBank accession number, infection state, and identity (%) with the reference genotype.

Species/Developmental stage	Place/Capture site	Blood source	BLAST score	*T. cruzi* infection	*T. cruzi* genotype
***R. prolixus*/Adult**	Cumaribo/Extradomicile	*Gallus gallus*	1 e-149, 99% FM205718.1	+	TcI
***R. prolixus*/Adult**	Cumaribo/Extradomicile	*Ceracris kiangsu*	8 e-147, 83% GU270284.1	+	TcI
***R. prolixus*/Nymphal**	Cumaribo/Extradomicile	*Ceracris kiangsu*	1 e-144, 83% GU270284.1	+	TcI

### 3.3 *Trypanosoma cruzi* infection in dogs

Most of the dogs sampled for this study live in houses not/enclosed by fences made of solid material, which allows animals to roam freely outside. All 465 dogs evaluated were creole breeds. The mean age of dogs was 3,65 ± 2,2 years (ranging from 6 months to 10 years old). Forty-nine dogs (10.53%, 95% CI=7.04-12.47%) were positive for *T. cruzi* by the ELISA and IFAT tests. The highest proportion of *T. cruzi* infection was in La Primavera, 23.28% (17/73), while the lowest infection was in Cumaribo, 8.4% (14/289) (**Table 1**).

### 3.4 *Trypanosoma cruzi* infection in synanthropic mammals and *Trypanosoma cruzi* genotyping

Twenty-two mammals were captured in and around the studied towns. *D. marsupialis* was the most abundant (Rodríguez-Monguí et al., 2019), followed by *Rattus rattus* (WHO, 2015)*, Proechymis semispinosus* (Chagas, 1909), and *Potus flavus* (Chagas, 1909). Of the 17 tested *D. marsupialis*, eight were positive for *T. cruzi* by haemocultures and molecular tools (**Table 1**). No infection was detected in the other mammals.

Discrimination between DTUs by SL-IR analysis revealed only the presence of DTUI in the samples of *R. prolixus*, *D. marsupialis*, and infected dogs.

### 3.5 Seropositivity in scholar students

A total of 3,425 kindergartens, primary and secondary students, were assessed. Two patients, twelve and twenty years old, residents of Cumaribo were positive, leading to an overall *T. cruzi* infection ratio of 0.06% (95% CI: 0.007-0.211) (**Table 1**).

## 4 Discussion

*Rhodnius prolixus* has been considered the primary vector species of *T. cruzi* in Colombia, especially in the Andean and Eastern Plains regions, where domestic populations have been linked to the Chagas disease domestic transmission cycle (Guhl and Ramirez, 2013). After extensive control efforts across this country, about half of municipalities considered at high risk were certified as free of *T. cruzi* transmission mediated by domestic *R. prolixus* (PAHO - Pan American Health Organization, 2019). However, in recent years, some authors have provided evidence of the participation of wild populations of this species, with less epidemiological relevance and involved in occurrences of an oral outbreak (Rendon et al., 2015; Zuleta-Duenas et al., 2017). These events have illustrated the species’ ability to participate in non-domiciliary transmission, suggesting the need to implement entomological surveillance instead of traditional programs of spraying domestic populations (Guhl et al., 2009; Cantillo-Barraza et al., 2021).

In this study, we highlighted some relevant facts in the Vichada Department: (i) the presence of *R. prolixus* exclusively in *A. butyracea* and *M. flexuosa* palms, (ii) the infection of this vector with TcI sylvatic, (iii) and reptiles as a food source, which support the presence of the sylvatic *T. cruzi* transmission cycle in this region of Colombia. This condition of non-domiciliation of this vector and the sylvatic cycle’s existence has been related to slow transmission to humans and moderate transmission to domestic dogs (Rendon et al., 2015). In concordance with this idea, this work showed low *T. cruzi* transmission to humans and domestic dogs in the Vichada department and extended area of Eastern Plains regions of Colombia without domiciliation and active participation of *D. marsupialis*.

The presence of *R. prolixus* in plantations of African oil palm *(E. guineensis)* in the Plains regions of Colombia and Venezuela has given rise to a novel epidemiological scenario for *T. cruzi* transmission (Cantillo-Barraza et al., 2021). In the present work, we did not evaluate the infestation of *R. prolixus* in African Oil Palms because these were located far from the assessed towns. However, this issue must be studied in the future because the municipalities of Santa Rosalia and La Primavera contain approximately 9,000 hectares of these crops (Fedepalma, 2020).

*Triatoma maculata* is one of the most widely distributed Triatoma species in northern South America, reported in Brazil (Luitgards-Moura et al., 2005), Colombia, Venezuela, Guyana, Suriname, French Guiana, and some Caribbean islands (Monsalve et al., 2016). This species is the most widely distributed secondary vector in Colombia after *Panstrongylus geniculatus* (Guhl et al., 2007)*. T. maculata* is a species with heterogeneous epidemiological relevance in Colombia and Venezuela. It has been found with high infection levels and an active role in *T. cruzi* transmission in the Caribbean region (Garcia-Alzate et al., 2014; Cantillo-Barraza et al., 2015; Hernandez et al., 2016). However, it has been reported without infection in other areas and is associated with birds (Guhl et al., 2007). In the study area, *T. maculata* was found without infection and associated with chicken coops. Therefore, it could be considered low epidemiological relevance for this area (Luitgards-Moura et al., 2005).

Furthermore, we reported a domestic dog infection frequency of 10.33% in the Vichada department. This result showed a significant intensity of *T. cruzi* transmission to this species. However, this value is lower than those reported in other Colombian regions, such as the Caribbean (70.1%), Andean (34%), and Eastern Plains regions (25.6%), where triatomines are present in the domestic and peridomestic areas (Cantillo-Barraza et al., 2015; Cantillo-Barraza et al., 2020a; Jaimes-Dueñez et al., 2020). The low intensity of *T. cruzi* infection reported here is congruent with reports in countries such as Panama, Brazil, and Venezuela, with enzootic transmission by triatomines species in palms (Calzada et al., 2006; Morocoima et al., 2010; Malavazi et al., 2020). Moreover, a similar situation was described by Rendon et al. in palm forests with sylvatic *R. prolixus* in the Casanare department, Colombia, with a regular sylvatic enzootic cycle.

In Colombia, different studies have demonstrated that domestic dogs play a role as synanthropic reservoirs that link domestic and sylvatic environments (Ramirez et al., 2013; Cantillo-Barraza et al., 2020b; Jaimes-Dueñez et al., 2020). TcI sylvatic in dogs supports this role in an area with sylvatic *R. prolixus.* In the Vichada department, the *T. cruzi* transmission has the extra domestic palms as microfoci; therefore, the infections could occur when domestic dogs enter the forest to hunt or accompany their owners. This absence of infestation may be related to the low intensity of transmission.

Additionally, *D. marsupialis* is the main reservoir in Colombia’s *T. cruzi* sylvatic transmission cycle (Rodríguez-Monguí et al., 2019). Recently, it has been suggested that this species play a relevant role as a synanthropic reservoir due to its behavior in some Colombian and Ecuador areas (Souto et al., 1996; Ocana-Mayorga et al., 2010; Betancourt-Echeverri et al., 2021). Our study showed that *D. marsupialis* was the most common sylvatic mammal with an infection level of 29.4% (5/17), reaffirming its importance in maintaining *T. cruzi* transmission in the study area. Moreover, the results support the species’ significance as a vehicle connecting the microfoci of *T. cruzi* transmission in forest palms with the villages where indigenes and colonist people of the Vichada department (Cantillo-Barraza et al., 2015; Rendon et al., 2015).

The serological evaluation results of the school-aged population in this study showed a low frequency of infection (0,06%, 95%CI: 0.007-0.211), suggesting a low level of contact among residents with *T. cruzi* transmission cycles. A similar situation was described by the serological results of school children in the Casanare department, where the infection was 1,25%, revealing an infrequent contact with the sylvatic cycle (Rendon et al., 2015; Olivera et al., 2019). The present study represents the most considerable collection effort and serological evaluation recorded in Colombia under field conditions after the National Study of Chagas between 1998 to 2002 (WHO. Grupo de trabajo científico, 2007). A total of 3,425 people throughout the Vichada department were included in this study, which allowed the inclusion of the entire indigenous population that voluntarily declared to participate. Our results contrast with the high *T. cruzi* prevalence reported in neighboring departments such as Casanare and Arauca (Llau et al., 2019; Olivera et al., 2019). However, epidemiological differences exist between these departments because, in the Vichada, the *R. prolixus* domiciliation was not registered in this study. It is important to highlight that this study was carried out during 2016-2017, data that can serve as an epidemiological baseline. Still, continuous surveillance must be established in this study area to keep the distribution and appearance of new cases updated.

In conclusion, the entomological, serological, and molecular evaluation of humans, domestic and synanthropic mammals showed that the Vichada department presents a non-domiciled and complete sylvatic cycle of *T. cruzi* transmission mediated by *R. prolixus* infesting palms of *A. butyracea* and *M. flexuosa.* These results show that *R. prolixus* wild populations have less epidemiological relevance than domiciled populations but require new approaches to control.

## Data availability statement

The datasets presented in this study can be found in online repositories. The names of the repository/repositories and accession number(s) can be found in the article/supplementary material.

## Ethics statement

With previous informed consent signed by both parents or legal tutors in the boarding school and following the requirement of the University of Antioquia (License 08-012-185), blood samples were collected from school-aged children, and assent forms were signed by the participants. Individuals over 18 years signed their consent forms/provided. Also, we had written permits from authorities of five indigenous ethnicities. The design and development of the study were carried out following the guidelines of the Declaration of Helsinki 2002 and the International Ethical Standards for Research in Humans and health research. All procedures were designed to reduce animal suffering. All owners were informed about the risks of Chagas disease, both for the human and canine populations. All animals were handled in strict accordance with the Colombian code of practice for the care and use of animals for scientific purposes, established by law 84 of 1989. Ethical approval (Act No 2223) for analyzing animal species was obtained from the animal ethics committee of Antioquia University.

## Author contributions

Conceptualization: OC-B and OT-C. Data curation: OC-B, MO, AM-J and SX. Formal analysis: OC-B, CS, AZ, RH, AM-J & OT-C. Funding acquisition: OC-B, CS, AZ and OT-C. Investigation: OC-B, CS, RH, MO, EG, AM-J and OT-C. Methodology: OC-B, CS, RH, MO, EG, SX, AM-J and OT-C. Project administration: OC-B and OT-C. Resources: OC-B, AZ and OT-C. Supervision: OC-B and OT-C. Validation: OC-B and OT-C. Writing – review & editing: OC-B, CS & OT-C. All authors contributed to the article and approved the submitted version.

## Funding

This research was supported by Universidad de Antioquia (UdeA), and SGR (Sistema General de Regalías), convenio interadministrativo 391 Gobernación del Vichada- Universidad de Antioquia.

## Acknowledgments

We are very grateful to the staff of the Health Secretary from the Vichada Department for the help collecting samples.

## Conflict of interest

The authors declare that the research was conducted in the absence of any commercial or financial relationships that could be construed as a potential conflict of interest.

## Publisher’s note

All claims expressed in this article are solely those of the authors and do not necessarily represent those of their affiliated organizations, or those of the publisher, the editors and the reviewers. Any product that may be evaluated in this article, or claim that may be made by its manufacturer, is not guaranteed or endorsed by the publisher.
